# An Overview of *In Vitro*, *In Vivo*, and Computational Techniques for Cancer-Associated Angiogenesis Studies

**DOI:** 10.1155/2020/8857428

**Published:** 2020-12-10

**Authors:** Heshu Sulaiman Rahman, Bee Ling Tan, Hemn Hassan Othman, Max Stanley Chartrand, Yashwant Pathak, Syam Mohan, Rasedee Abdullah, Noorjahan Banu Alitheen

**Affiliations:** ^1^Department of Physiology, College of Medicine, University of Sulaimani, 46001 Sulaymaniyah, Iraq; ^2^Department of Medical Laboratory Sciences, College of Health Sciences, Komar University of Science and Technology, Chaq Chaq Qularaesee, 46001 Sulaymaniyah, Iraq; ^3^Department of Nutrition and Dietetics, Faculty of Medicine and Health Sciences, Universiti Putra Malaysia, 43400 UPM Serdang, Selangor, Malaysia; ^4^Department of Pharmacology and Toxicology, College of Pharmacy, University of Sulaimani, 46001 Sulaymaniyah, Iraq; ^5^DigiCare Behavioral Research, Casa Grande, Arizona, USA; ^6^College of Pharmacy, University of South Florida, Tampa, USA and Adjunct Professor at Faculty of Pharmacy, University of Airlangga, Surabaya, Indonesia; ^7^Substance Abuse and Toxicology Research Center, Jazan University, Jazan, Saudi Arabia; ^8^Department of Veterinary Laboratory Diagnosis, Faculty of Veterinary Medicine, Universiti Putra Malaysia, 43400 UPM Serdang, Selangor, Malaysia; ^9^Department of Cell and Molecular Biology, Faculty of Biotechnology and Bio-Molecular Sciences, Universiti Putra Malaysia, 43400 UPM Serdang, Selangor, Malaysia; ^10^Institute of Bioscience, Universiti Putra Malaysia, 43400 UPM Serdang, Selangor, Malaysia

## Abstract

Angiogenesis is a crucial area in scientific research because it involves many important physiological and pathological processes. Indeed, angiogenesis is critical for normal physiological processes, including wound healing and embryonic development, as well as being a component of many disorders, such as rheumatoid arthritis, obesity, and diabetic retinopathies. Investigations of angiogenic mechanisms require assays that can activate the critical steps of angiogenesis as well as provide a tool for assessing the efficacy of therapeutic agents. Thus, angiogenesis assays are key tools for studying the mechanisms of angiogenesis and identifying the potential therapeutic strategies to modulate neovascularization. However, the regulation of angiogenesis is highly complex and not fully understood. Difficulties in assessing the regulators of angiogenic response have necessitated the development of an alternative approach. In this paper, we review the standard models for the study of tumor angiogenesis on the macroscopic scale that include *in vitro*, *in vivo*, and computational models. We also highlight the differences in several modeling approaches and describe key advances in understanding the computational models that contributed to the knowledge base of the field.

## 1. Introduction

Normal physiological angiogenesis is the development of new blood vessels by endothelial cell proliferation and outgrowth rom the existing vasculature [1]. Thus, new blood capillary formation is important to provide tissue oxygenation, burn metabolic substrates, and enhance energy production especially during wound healing, menstruation cycle, and pregnancy [2]. However, dysregulation of normal physiological angiogenesis plays an important role in the progression and initiation of numerous disorders including age-related macular degeneration and malignant tumors [3].

Within the tumor mass, the availability of nutrients is limited by competition among actively proliferating cells, and the diffusion of metabolites is impeded by high interstitial pressure [4]. As a result, tumor cells induce the development of a new blood supply from the preexisting vasculature, and this affords tumor cells the ability to survive and propagate in a hostile environment [5]; hence, angiogenesis is crucial for the initiation, promotion, and metastasis [3]. Generally, angiogenesis nourishes the tumor mass during hypoxia. This process is vitally important in cancer cell survival and metastasis and is highly dependent on key angiomodulatory factors [6].

**Figure 1:**
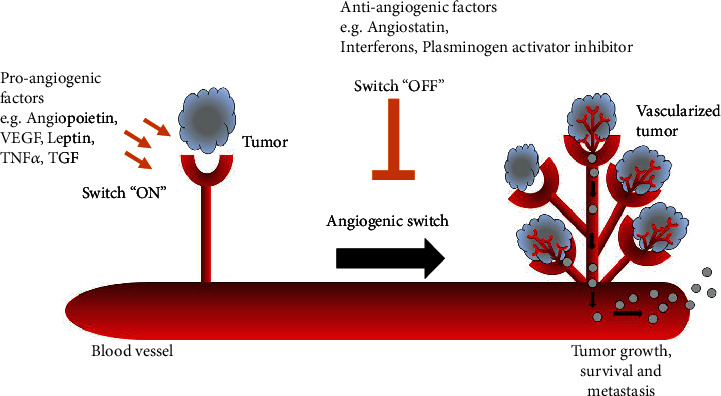
The angiogenic switch in carcinogenesis. The switch is controlled by the balance between pro- and anti-angiogenic factors. Neovascularization supplies essential nutrients and oxygen to the growing tumor, and promotes the tumor survival and metastasis.

It is well-recognized that the “angiogenic switch” (Figure 1) is “off” when the effect of proangiogenic molecules is balanced by antiangiogenic molecules and is “on” when the net balance is tipped in favor of angiogenesis. Several signals activate this switch such as genetic mutations, immune/inflammatory response, mechanical stress (pressure generated during cell growth), and metabolic stress (low pH or low *p*O_2_) [7]. Among the angiogenic molecules, angiopoietin, and vascular endothelial growth factor (VEGF) family members have a prominent role in angiogenesis [8].

While some models have studied signaling phenomena at the level of cell membrane-bound receptors [9], others have focused on microvascular network remodeling at the level of whole tissues [10]. Here, we review the standard models for the study of cancer angiogenesis. This review summarizes the types of *in vitro*, *in vivo,* and computational models of angiogenesis. We also highlighted the differences in several modeling approaches and described the key advances in understanding computational models that contributed to the knowledge base of the field.

## 2. Angiogenesis Assays

### 2.1. *In Vitro* Techniques

*In vitro* experiments are a precious means for investigating and evaluating the effects of angiogenesis and antiangiogenic agents. They can be rapidly and simply conducted using fundamental methods and quantitative measurements. These techniques are basic for determining cellular, biochemical, and molecular mechanisms of action of any new anticancer product or therapy. Despite analysis processes that contribute to angiogenesis mechanisms including endothelial migration, proliferation, sprouting, branching, differentiation, and lumen formation, concomitant complex cell-cell and cell-extracellular matrix (ECM) interactions can also be performed [11–13]. The principle of *in vitro* assays includes the proliferation of endothelial cells, migration, differentiation, and coculture with mural cells and fibroblasts and vessel outgrowth from organ cultures.

#### 2.1.1. Types of Endothelial Cells

Mature endothelial cells have been the most commonly used in such studies. They can be collected from various species and sources, including bovine, canine, porcine, and human. Cultured endothelial cells have similarities to *in vivo* angiogenic endothelial cells [14]. Endothelial cells can be harvested either from large vessels, such as an umbilical vein, jugular vein, and aorta, or from microvessels, such as the dermis [15]. The most common macrovascular endothelial cells in use currently are human umbilical vein endothelial cells (HUVECs) and human arterial endothelial cells (HAECs). It is well known that angiogenesis only occurs in microvascular beds, but because of their availability and low cost of maintenance, most frequently, macrovascular cells are preferred in the laboratory setting [16]. HUVECs are easily isolated, cultured, highly propagated, and prone to form capillaries, which render it to be the common one for *in vitro* angiogenesis evaluation. Likewise, big vessels such as the aorta are the common source HAECs, which are subsequently suggested for use in testing pathological processes, such as thrombosis, atherosclerosis, and hypertension [17].

The most popular types of microvascular endothelial cells used *in vitro* assays either are derived from human organs and are referred to as human microvascular endothelial cells (HMVECs), such as human dermal microvascular endothelial cells (HDMECs) and human brain microvascular endothelial cells (HBMECs), or are derived from animal sources, such as bovine aortic endothelial cells (BAECs) and porcine aortic endothelial cells (PAECs) [18] [19]. HMVECs are the cells of choice for tumor neoangiogenesis studies because they are the courier of endothelial cells of the surrounding tumor tissues. The cell that is used for cell culture assays of angiogenesis should be selected as closely as possible to be similar to the tissue of interest [20]. Due to their ease of isolation and culture, HDMECs are considered the second most commonly used cell type in studies of endothelial cell assembly [5].

#### 2.1.2. Endothelial Cell Proliferation Assays

Cell proliferation assays are commonly used because they are tremendously reproducible and easy to work with and generate specific and particular data. These studies should be carried out on cells that are passages 3 to 6. The convergence of endothelial cells must be lower than 70% as the proliferative activity is reduced when the cells reach confluence [21]. Different methods are available for measuring the proliferation of cells, including cell counting and DNA synthesis analysis. It is strongly recommended that at least 2 or more methods are used to obtain the most dependable and accurate data with proliferation assays. Direct determination of the cell number can be used to measure the proliferation of the cells. Most probably in the majority of the tests, a specified number of endothelial cells are plated and allowed to proliferate in a specific period. Later, increases in the number of cells are measured by direct cell counting using a hemocytometer or a Coulter counter. More recently, the Vi-cell counter is used for this purpose, which measures both cell number and viability, but this technique is highly vulnerable to errors, as well as it is considered a time-consuming method [22, 23] compared to other previously used cell viability assays such as tetrazolium reduction assay (MTT, MTS, XTT, WST-1, and CCK-8) and resazurin reduction assay [24].

Currently, DNA synthesis techniques are used as an alternative measure of cell proliferation, in which scintillation counters are used to measure the incorporation of [^3^H]thymidine into the DNA of the cells. In this method, the amount of radioactivity is directly proportional to the synthesis of new DNA [25]. Another approach that competes with thymidine incorporation into the DNA to assess DNA synthesis is by using bromodeoxyuridine (BrdU). The incorporated BrdU can be detected by immunocytochemistry or by using ELISA techniques. More recently, the expression of proliferating cell nuclear antigen (PCNA) can be measured in cells using immunocytochemical analysis [26].

#### 2.1.3. Endothelial Cell Migration Assays (Cell Invasion Assays)

Migration of endothelial cells into the perivascular stroma can occur through the degradation of the basement membrane in response to angiogenesis inducing factors, such as vascular endothelial growth factors (VEGF). The different available methods for measuring endothelial cell migration include the Boyden chamber method (transfilter assay), scratch wound method (scrape wound), and phagokinetic track method [27].

The most commonly used technique to determine endothelial cell migration is a modification of the Boyden chamber, which is currently called transfilter assays [28]. This method can differentiate between random motility (chemokinesis) and directional migration towards a stimulus (chemotaxis), in which endothelial cells are seeded on the top of a cell-permeable filter (polycarbonate or polypropylene) [29] that allows only active passage of the cells to migrate towards the test angiogenic factor that is positioned in the bottom chamber. Furthermore, the filter may be coated with matrix components, such as collagen, fibronectin, laminin or a reconstituted matrix, such as Matrigel, as an effort to simulate the *in vivo* microenvironment [30]. Measurements are applied by counting cells that have migrated on the lower side of the filter. This method is characterized by being highly sensitive, high reproducibility, and a short duration of time for performance (4-6 hours). Cell motions during experiments are very difficult to observe as it is a time-consuming and not accurate way to count cells by the naked eye [31]. Using crystal violet to stain migrating cells then washing out the stain is also another method of choice for migration measurement in the modified Boyden chamber. Since the concentration of the used stain normally correlates with the amount of migrated cells, the migration can be measured spectrophotometrically using the ELISA plate reader [32]. Another way of counting the cells is by fluorescent labeling of the endothelial cells and the use of a filter made of polyethylene terephthalate, a light-shielding material. Only the migrating endothelial cells are visible to the fluorescent plate reader and can be counted accordingly [33].

There is also another simple way to quantify the migration of endothelial cells termed scratch wound assay. This method is dependent on the ability of endothelial cells to fill a cleared area that has been scratched on a confluent monolayer of endothelial cells [34]. Usually, a pipette tip or cell scraper is used to create a clearing for an area of a wound at the monolayer. The rate and extent of endothelial cell migration towards the scratched area can be quantified either by measuring the time required to close the wound area or by measuring the distance moved by the endothelial cells or the area enclosed by the endothelial cells. Using image processing, results can be quantitatively analyzed as a free version ImageJ [35]. This assay is simple, quick, and inexpensive and can be applied for high-scale screening. The main disadvantages of this method are the difficulty to create scraped areas of equal size and the variability between experiments due to the difference in the degree of initial cell confluence [36]. Modification of scratch wound assay is done via using a ring barrier and Teflon fence. In the latter, a Teflon fence is used to restrict the endothelial cells to a region of a well and then allowing it to propagate until confluence. Following fence removal, migrating cells are fixed and counted at specific times [37]. An advanced microfluidic version of the scratch wound assay, in which the artificial wound is precisely created using a laminar flow of trypsin solution, was introduced to study the effect of shear stress on cell migration. Moreover, the phagokinetic track method is implanted to determine the direct motility of the cells and directional effects on cell movement [38].

#### 2.1.4. Endothelial Cell Differentiation Assays

The differentiation and development of capillary-like vessels are the characteristic features of the later stages of angiogenesis. Endothelial cell differentiation can be assayed using the basic tubule formation method in which a specific layer of the gel matrix, which may be collagen, fibrin, or Matrigel, is used for the plating of its endothelial cells [39]. This step mimics the attachment, migration, and differentiation of endothelial cells into the production of tubule-like structures [26]. *In vitro*, an organization of endothelial cells into tube-like structures has been studied for decades on 2D-coated plates in two-dimensional assays and on 3D gels in three-dimensional assays [40]. The 2D assays are characterized by the formation of cell tubules in the horizontal plane. In the initial assay invented by Pepper et al., endothelial cells are seeded as a monolayer onto the surface of fibrin gels or collagen, and some endothelial cells plague the matrix to form tube structures. The tubule production is observed over a 4 to 24-hour period in the presence of the tested compounds and recorded using a digital camera [41]. Alternatively, two layers of collagen can be used to seed endothelial cell monolayer between them [42]. The differentiation of endothelial cells to form tubules depends upon the type of matrices selected for the assay [43]; therefore, it is essential to perform the assays using more than one matrix to corroborate the action of several test substances. The variables that can be used to assess the extent of tubule formation include number, length, and area of tubules that can be measured manually or via image analysis programs. This method is quick, reliable, and easy to set up and is usually used to test novel compounds for pro- or antiangiogenic effects. One limitation of this method is that some cultured cells of nonendothelial origin, such as fibroblasts or cancer cells, may also respond to Matrigel by forming tube-like structures [44]. It is important to point out that actual blood vessel formation is much better than mere tube formation as the two are not equivalent. Furthermore, the aortic ring assay for simulation of *in vivo* angiogenesis is the most reliable approach to study the role of both pericytes and endothelial cells, [45] since it reduces the period taken for the vessel formation under *in vivo* condition. In this approach, the isolated rat aorta is cut into segments that are placed in a culture of Matrigel. Cells are then monitored over the next 7-14 days for the growth of endothelial cells. Quantification is achieved by the measurement of the length and abundance of the resulting vessel-like extensions. Additionally, the coculturing of the endothelial cells with stromal cells which can be fibroblasts or smooth muscle cells is an alternative method for the formation of the tubule [46]. These assays are characterized by having the supporting cells that secrete a matrix for the endothelial cells to differentiate accordingly. However, these assays are time-consuming and need about 2 weeks; then, the results will give the proliferation measurement as well as cell differentiation [47].

The extracellular matrix and cell-to-cell interactions are not present in two-dimensional (2D) cell culture models. The three-dimensional (3D) cell culture system has more advantages when compared to 2D culture in terms of reflecting *in vivo* conditions at many levels, such as cell movement, cell morphology, cell-cell adhesion, and cell polarity [48]. The 3D flat-shaped assay including both 3D basement membrane and cell sheet assays more closely mimics *in vivo* angiogenesis where tubule formation occurs in vertical and horizontal planes [49]. The 2D basement membrane assay described above is easily modified to become a 3D scaffold model simply by increasing the thickness of the basement membrane or by overlaying an additional layer. The 3D basement membrane assay has many advantages compared to the 2D assay. It enables endothelial cells to form not only in capillary-like structures but also as lumens. The migration of endothelial cells can be observed and analyzed easily in both horizontal and vertical directions. However, 3D assays require a longer time to run (5-15 days), are difficult to view and quantify, and require histological techniques or confocal microscopy. Care should also be given to the width of the matrix that might result in additional difficulties in the diffusion of nutrients and oxygen [49, 50].

In an alternative 3D assay (3D spherical-shaped assay or microcarrier or microtissue assay), microcarrier beads are used for the growth of endothelial cells into a confluency rate, which is then implanted into fibrin gels. Consequently, the migration of endothelial cells that form capillary-like structures has been observed and quantitatively analyzed. Microcarriers can be made of dextran, collagen, cellulose, plastic, glass, and fibrin with a diameter of 100-400 *μ*m suitable for cell attachment [51]. The advantage of this model is that it can avoid the endothelial cell detachment problem that is often encountered in 2D models. One disadvantage of this method, however, is the possibility that microcarriers may descend to the base of the gel. Another challenge that is common to all the 3D assays is the breadth of the gel, which has to be relatively thinner to permit the dispersion of oxygen and nutrients; otherwise, it can lead to proliferative cell deaths [52].

Microtissue is a spherical aggregation of the cells that can be formed using varying techniques [53, 54]. Microtissue sizes can be controlled by changing the cell number and should yield structures of 100 500 *μ*m in diameter. When the diameter of the microtissue is more than 200 *μ*m, the diffusion of many molecules, especially oxygen, is limited leading to hypoxia inside the microtissue core. Microtissue assay is easy and inexpensive, allows flexibility in cell-type composition, and is applied to high-throughput drug screening. However, it is difficult to observe and analyze cell behavior in microtissue, because of its spherical shape and thickness [50].

#### 2.1.5. Endothelial-Mural Cell Coculture Assays

There is a preponderance of *in vitro* techniques that concentrates on endothelial cell proliferation, migration, and differentiation. Similarly, other cell types are also important, such as supporting cells that may be found in smooth muscle cells, pericytes, fibroblasts, and tumor cells. On the other hand, the extracellular matrix (ECM) and/or basement membrane and circulating blood may also play a major role. Various efforts at coculturing endothelial cells with different cell types are performed, but there is no *in vitro* assay currently available that covers all the components of this complex process [55].

Furthermore, direct contacting assays may be used to evaluate the straight effects of one cell type upon another [56]. The simplest direct contacting coculture assays include direct plating of the cells or permitting one cell type to adhere first and then seeding the second cell type on top. To study the effects of one cell type on the proliferation of another cell type, one is expected to label at least one population before the seeding to be quantified [57]. These assays have been used to determine the effects of endothelial cells on mesenchymal cell differentiation as they are simple and easy to analyze, but they lack the effect of paracrine factors that can be released by one cell type. This limitation can be overcame by using noncontact coculture assays. Similar to the tubule formation assays, endothelial-mural cell coculture assays can be prepared in 3D where endothelial and mural cells are sandwiched between layers of Matrigel and tubules that are placed into the Matrigel [58].

Regarding the endothelial-tumor cell coculture system, capillary-like structures were induced in fibrin gel in which collagen gels containing fibroblasts and/or human prostate adenocarcinoma cells (PC-3) were sandwiched together. In the presence of collagen-embedded fibroblasts, angiogenesis occurred, while endothelial cells did not survive when only PC-3 cells were embedded in collagen. In contrast, when PC-3 cells were combined with fibroblasts in collagen gel; an enhanced formation of capillary-like structure formation was noted, particularly using the FGF-2-supplemented medium [59]. Additionally, a 3D human cell culture system was constructed from colon cancer cells cocultured with normal fibroblasts or cancer-associated fibroblasts (CAFs) in collagen I gel that resulted in the interaction of colon cancer cells with stromal fibroblasts which induced different highly relevant cancer expression profiles which mediated paracrine interactions in the tumor microenvironment and validated the influence of these molecular targets during tumor growth and invasion in the supporting stroma [60]. Also, the endothelial-hepatocellular carcinoma (HCC) cell (HepG2) coculture system was induced in which endothelial cells were differentiated to form tubule networks [47].

Although *in vitro* assays are most beneficial for screening the effectiveness of new drugs, precautions should be taken in analyzing the results, especially where differences between the lineages of these cells can contaminate results. It is therefore necessary to select the technique and cell types that are most likely the angiogenic ailment that is being studied [61]. More specifically, human dermal microvascular endothelial cells should be used to study psoriasis, whereas breast microvascular endothelial cells could more appropriately be used to study mammary gland adenocarcinoma. Extracellular matrices and supporting cells that are derived from smooth muscle cells, mural cells, and fibroblasts should be available when the endothelial cells are cultured [62]. The culture conditions should be adjusted to closely imitate the *in vivo* situation. Multiple assay types and conditions should be used to validate the results using and/or comparing *in vitro* effects against results that are observed with *in vivo* assays.  Table 1 shows the *in vitro* techniques of angiogenesis [63].

### 2.2. *In Vivo* Techniques

#### 2.2.1. Quantitative Determination of Tissue Blood Flow Rate (BFR)

A functional blood vessel network is formed as a result of the maturation phase of angiogenesis. Measuring the BFR through the network contributes to a better understanding of the angiogenic process. The BFR will impact the efficiency of the delivery of oxygen, nutrients, and drugs to the surrounding tissues. These parameters are critical for treatment effects detected during the treatment of malignancies. The BFR is more sensitive and acquires an appropriate pharmacodynamic endpoint when determining the efficiency of vascular distracting approaches in cancer treatments [64].

The BFR is the process of delivering the arterial blood into the capillary beds within a scrupulous group of tissues. The BFR is measured in units of milliliters of blood per gram of tissue per minute (mL·g^−1^·min^−1^) or is measured in units of milliliters per unit volume of tissue (mL·mL^−1^·min^−1^). There are many investigational techniques used to measure the blood volume of tissues and the measurement of red blood cell (RBC) velocity (*μ*m·s^−1^) in individual capillaries facilitated by intravital microscopy. The most reliable and authentic approach to measure the BFR is to measure the rate of delivery of an agent carried to the tissue by blood flow. In this approach, a contrast agent (an inert compound) is injected into the blood circulation, where its input function and tissue response function are measured by its concentration time course in arterial blood, together with the kinetics of its uptake in tissue, respectively. Finally, the BFR is measured by using a mathematical model related to the tissue response function to the input function [65].

Generally, 2 main types of contrast agents are applied. The first one is the radioactive type that reveals detectable concentrations in tissue and is measured by gamma or scintillation counting or an external advanced technology imaging system such as a positron emitter for positron emission tomography can be used. The second contrast agent is also appropriately used for external magnetic resonance imaging, computed tomography scan, or ultrasound imaging. Among these types of contrast agents, radioactive agents are superior and have an advantage over external contrast agents. Indeed, they do not interfere with physiological processes. Most notably, they do not require advanced imaging technology [66].

Some common methods/assays are available for measuring blood perfusion parameters, yet not all of them provide a fully quantitative measurement of BFR. Among them, Laser Doppler Flowmetry (LDF) is a technique used for microvascular blood perfusion assessment. It provides a means of estimating relative changes in RBC velocity. Doppler refers to the frequency shift that arises in the light that has been scattered by moving RBC (a measure of average RBC velocity). On the other hand, variation in RBC velocity may not accurately reproduce the stated changes in flow rate [67].

More recently, fluorescent DNA-binding staining Hoechst 33342 and certain carbocyanine staining have been applied to measure perfused vascular volume as a fraction of the total tissue volume rather than BFR. In this method, tissues precede excision after many cycles of circulation. Finally, functional vessels appear as fluorescent halos after intravenous injection of the radioactive dye. Nevertheless, a measure of vascular function is valuable under many conditions, but it is unable to discriminate between perfused vessels with different flow rates and lacks appropriate sensitivity. [68]

The methods that are based on Eq. 1 might be used to calculate the flow rate when the contrast agents are confined to the bloodstream [69]. So far, these methods have had difficulty in practice because the operation takes only a few seconds, requiring a highly sensitive and quick technique for measurement. A special category of contrast agents, which may be radioactive or colored microspheres with a 15-5 *μ*m diameter, is confined to the bloodstream since they should be fascinated on the first pass through the tissue. Care should be taken with this technique, and one should assure proper mixing of microspheres in the arterial blood (exigent in mice, for instance). Also, in the targeted tissue site, adequate microspheres should be located to obtain statistical validity. However, in the case of tumors, one should make sure that determination and correction for microspheres are executed with precision because of potential problems of recirculation of microspheres that can arise due to a lack of capture in large-diameter vessels [70].

#### 2.2.2. In Vivo Matrigel Migration and Angiogenesis Assay

The formation of new blood vessels from preexisting vessels is the standard process of angiogenesis. This process involves developmental processes, wound healing, migration, and growth of pathologic conditions like cancer and vasculitis. Because angiogenesis plays a critical role, a rapid *in vivo* method that determines the angiogenic potential of compounds is advantageous for augmenting *in vitro* findings [71].

The marine Matrigel plug assay is a quantitative method that is beneficial for measuring both angiogenesis and antiangiogenesis. Matrigel, an extract of the Engelbreth-Holm-Swarm tumor, is composed of basement membrane components and liquefies at 4°C as it forms a gel while warming to 37°C [72]. After plating on human umbilical vein endothelial cells (HUVECs), it ensures differentiation into capillary-like tube structure *in vitro* [73]. However, *in vivo* Matrigel in injected form is utilized as a mixture that includes prospective angiogenic compounds or it can be used alone. When injected subcutaneously (SC) into the ventral region of mice, it solidifies and forms a “Matrigel plug” concerning its temperature characteristics. When Matrigel mixed with known angiogenic compounds, such as basic fibroblast growth factor (bFGF), and injected into the mouse, stimulation of the endothelial cell migration into the plug is observed. These plug-forming vessels contain erythrocytes (indicating functional capillaries) and the von Willebrand factor (factor VIII). The observation of the angiogenesis level of these plugs is done by embedding and sectioning in paraffin and staining by Masson's Trichrome: the Matrigel stains blue, while the endothelial cell/vessels stain red. It appears that factor VIII stains positive in these capillaries [74, 75].

In the case of unsupplemented Matrigel, only a few cells will invade the plug. It is noted that yellowish plugs are formed when strong angiogenic compounds are used, with the intention that preliminary indications of activity can be made at the time when plugs are removed from the mice. The essay gives the best of results when testing putative antiangiogenic compounds so that Matrigel can be premixed with bFGF (angiogenic compound and potent inducer of neovascularization) with the addition of the test substances. Therefore, inducing the activity of bFGF for the formation of vessels in the Matrigel plug is inhibited by anti-angiogenic substances. The plugs are comparatively colorless when they are removed from the mouse, and only a few endothelial cells are viewed by Masson's Trichrome staining. Due to the bFGF powerful vascular response in the plug, the measurement of hemoglobin content is made possible with the Drabkin assay [76]. For the quantification of plasma volume in the plugs, fluorescein isothiocyanate dextran (145,000-200,000 MW) is injected intravenously into the tail vein [77]. Otherwise, fluorescent microscope and imaging software can be used to visualize and quantify the vessels [78].

An important consideration should be kept in mind about some inconsistent observations that may be found while using either of the above assays. In one observation, differences were observed in the mice and in the basement membrane preparations that could be affecting the levels of the surrounding of blood vessel formation. Also, the gender and age of the mice can cause observable inconsistencies in results. Vessel formation is reduced in young mice (<6 months old) when compared to older mice (>12-24 months old) [79]. Variability in results is also observed when Matrigel is applied at different sites in the mouse. If Matrigel is injected into the dorsal surface of the animal, a reduced angiogenic response is observed, whereas the ventral surface of the mouse in the groin area close to the dorsal midline has been proved to be one of the best areas for an angiogenic response. Despite such potential problems, this assay is considered the best for the rapid screening of potential angiogenic and antiangiogenic compounds [71].

#### 2.2.3. The Corneal Pocket Assay

In the development and evaluation of drugs that could cause suppression of angiogenesis, there is a critical need for continuous monitoring of angiogenesis *in vivo*. Hence, intensive efforts should be made to design an ideal animal model for better quantitative analysis of *in vivo* angiogenesis [80].

The corneal assay is composed of the angiogenesis inducer (i.e., tumor tissue, cell suspension, and growth factor) which is implanted into a micropocket produced in the cornea thickness to induce angiogenesis by vascular outgrowths from peripherally located limbic vasculature. One advantage of this relatively simple assay is that it shows excellent reproducibility compared to other *in vivo* assays. Another advantage lies in the fact that since the cornea normally lacks blood vessels, the background is kept minimal in the assay [81].

The corneal assay was first described by Gimbrone et al. in their research that was conducted in New Zealand on white rabbits. However, the original essay was modified to allow the implantation of multiple samples, including cell suspensions and tissue fragments. Its choice was justified by having the above-mentioned advantages, namely, the absence of a vascular pattern and easier manipulation and monitoring of the neovascular growth. For many years, this technique has been widely used and has been considerably modified to fulfill different experimental objectives: characterization of angiogenesis inducers, evaluation of angiogenesis inhibitors, interaction between different factors, and the study of cellular, biochemical, and molecular mechanisms of angiogenesis [82].

#### 2.2.4. Chick Embryo Chorioallantoic Membrane (CAM) Assay

The CAM is an extraembryonic membrane composed of a multilayer epithelium; the ectoderm at the air interface, mesoderm (stroma), and endoderm at the interface with the allantoic sac [83]. Also, it contains extracellular matrix proteins such as fibronectin, laminin, collagen type I, and integrin *ανβ*з that collectively mimic the physiological cancer cell environment [84, 85].

CAM assay has been widely used to study angiogenesis, tumor invasion, and metastasis of colorectal, prostate, and brain cancers, and it is considered a very useful *in vivo* model for screening of potential novel therapeutics [86, 87]. Regarding ovarian cancer angiogenesis, a CAM assay protocol was developed to monitor the metastatic properties of OVCAR-3, SKOV-3, and OV-90 cells and to study the effect of potential therapeutic molecules *in vivo*. The results from the CAM assay were consistent with cancer cell motility and invasion observed in *in vitro* assays, and consequently, the CAM assay reflected a robust and cost-effective model to study ovarian cancer cell metastasis [86].

#### 2.2.5. Other In Vivo Models

In addition to the *in vivo* techniques discussed here, other interesting and exciting models have recently been reviewed by Eklund et al. and have emerged as important research in angiogenesis. These include mouse syngeneic models, human xenografts, transgenic mouse models, and mutagenesis-induced mouse models. Other preclinical and clinical models with high resolution and deep imaging techniques were developed to monitor the blood of the tumor in real-time and accompanying cellular events that coincided with pharmacodynamic endpoints [88].  Table 2 shows the *in vivo* techniques of angiogenesis.

### 2.3. Computational Methods

Developing mathematical models to describe tumor growth, metastatic spreading, and specific tumor biologic pattern, such as neoangiogenesis, is a rising trend in oncology. A wide variety of mathematical models have been made available, from multiscale and highly mechanistic models to simplified, phenomenological models [89]. Since the late 90s, several models have focused on describing neoangiogenesis mostly as part of *mechanistic* and *theoretical* approaches [90]. Additionally, neoangiogenesis can be described following a *descriptive* approach, thus yielding more simplified *phenomenological* models [91]. Phenomenological models offer the advantage of simplicity, thus providing better identification of parameters. The downside of this approach is a lack of basic understanding of the underlying mechanisms. However, such a simple model approach can be easily plugged in with an efficacy compartment, thus allowing one to predict how drugs will impact neovasculature and therefore on tumor growth. Consequently, mathematical modeling of neoangiogenesis can be used as an *in silico* tool to simulate the impact of a variety of drugs and regimen, thus allowing virtual screening of new compounds or comparisons between different regimens. For instance, the impact of metronomic therapy on tumor angiogenesis has been modeled following a phenomenological approach [92, 93]. Such a model was next used to determine the best dosing and scheduling with metronomic vinorelbine to maximize its antiangiogenic properties [94, 95]. Other PK/PD models have also been proposed as a means to study the direct impact of drugs on tumor vasculature, although they yet remain theoretical [96, 97].  Table 3 shows the computational techniques of angiogenesis.

Angiogenesis requires a crucial balance between antiangiogenic and proangiogenic factors, and a shift in these factors can subsequently result in pro- or antiangiogenic effects [98]. Current assays for angiogenesis are too complex to be practical for drug screening. Recent efforts have produced *in vitro* microfluidic cell culture models that apply physical and biochemical stimuli within a 3D hydrogel scaffold integrated into the channels and offer a viable solution for monitoring cellular behaviors in response to drugs [99, 100]. However, an *in vivo* model still considered the preferable assay for drug screening such as the zebrafish especially for screening small molecules that affect blood vessel formation. Blood vessel patterning is highly characteristic in the developing zebrafish embryo, and the subintestinal vessels can be stained and visualized microscopically as a primary screen for compounds that affect angiogenesis [101].

## 3. Conclusion

Studies in the past few decades have tremendously advanced our understanding of the angiogenesis process and provided further insight into both the function and genetics of this process, which impacts blood vessel formation in disease and health. Through the contribution of growth factor immobilization, VEGF and VEGF-mediated signaling have all been studied *in vitro* (and, to a more limited extent, *in vivo*); however, the combined regulation of these cues within the context of the human study has yet to be fully studied. Computational models provide a key tool to study the combined effects of many forms of regulation within a single framework between human patients and model systems. Although computational models have notable limitations, further advances in the development of this model will undoubtedly come rapidly, given the intensity of ongoing research efforts. Taken together, a combination of *in vitro* studies, analytical and functional *in vivo*, and predictive computational models of angiogenesis can help to understand and identify better the angiomodulatory factors and how it controls angiogenesis in disease and health.

## Figures and Tables

**Table 1:** *In vitro* techniques of angiogenesis.

*In vitro* techniques	Type of methods	Biological scope	Assay reliability (quantitative or qualitative)	Pros/cons	Reference
Endothelial cell proliferation assays	Cell counting	A specified number of endothelial cells are plated and allowed to proliferate in a specific period of time. Increases in number of cells are measured by direct cell counting using hemocytometer or a Coulter counter	Measure both cell number and viability	Highly vulnerable to errors and time-consuming	[22, 23]

DNA synthesis	—	Scintillation counters are used to measure the incorporation of [^3^H]thymidine into the DNA of the cells	Measures cell proliferation	Highly vulnerable to errors	[25]
	Bromodeoxyuridine (BrdU)	The incorporated BrdU can be detected by immunocytochemistry or using ELISA techniques	Measures cell proliferation	Highly vulnerable to errors	[26]

Endothelial cell migration assays (cell invasion assays)	Boyden chamber method (transfilter assay)	Endothelial cells are seeded on the top of a cell-permeable filter (polycarbonate or polypropylene) that allows only active passage of the cells to migrate towards the test angiogenic factor that is positioned in the bottom chamber	Cell counting	Highly sensitive, high reproducibility, short duration of time for performance (4-6 hrs), time-consuming, and not accurate way to count cells	[21, 29, 31]
	Modified Boyden chamber	Using crystal violet to stain migrating cells then wash out the stain	Measure spectrophotometrically using ELISA plate reader	The concentration of the used stain normally correlates with the amount of migrated cells	[32]
	Fluorescent labeling	Fluorescent labeling of the endothelial cells and the use of filter made of polyethylene terephthalate, a light-shielding material	Cell counting	Migrating endothelial cells are visible to the fluorescent plate reader	[33, 102]
	Scratch wound (scrape wound) method	A pipette tip or cell scraper is used to create a clearing for an area of a wound at the monolayer	Quantify the migration of endothelial cells	Simple, quick, and inexpensive and can be applied for high-scale screening Difficult to create scraped areas of equal size and the variability between experiments due to the difference in degree of initial cell confluence	[35, 36, 103]
	Modification of scratch wound assay (Teflon fence)	To restrict the endothelial cells to a region of a well and then allow to propagate until confluence	Cell counting	Following fence removal, migrating cells are fixed and counted at specific times	[37]
	Advanced microfluidic version of the scratch wound assay	To study the effect of shear stress on cell migration	Measure cell migration	Artificial wound is precisely created using a laminar flow of trypsin solution	[104]
	Phagokinetic track method	To determine the direct motility of the cells and directional effects on cell movement	Determine direct motility	Determine direct movement of cells	[21, 38]

Endothelial cell differentiation assays	2D-coated plates in two-dimensional assays	Formation of cell tubules in the horizontal plane	To assess the extent of tubule formation include number, length, and area of tubules that can be measured manually or via image analysis programs	Quick, reliable, and easy to set up Some cultured cells of nonendothelial origin, such as fibroblasts or cancer cells, may also respond to Matrigel by forming tube-like structures	[41, 44]
	Aortic ring assay	Isolated rat aorta is cut into segments that are placed in a culture of Matrigel. Cells are then monitored over the next 7-14 days for growth of endothelial cells	Quantification is achieved by measurement of the length and abundance of the resulting vessel-like extensions	The most reliable, reduces time taken for the vessel formation under *in vivo* condition	[45, 105]
	Coculturing of the endothelial cells with stromal cells (fibroblasts or smooth muscle cells)	Characterized by having the supporting cells that secrete a matrix for the endothelial cells to differentiate accordingly	Measure cell proliferation and cell differentiation	Time-consuming	[46, 106]
	3D basement membrane assay	Enables endothelial cells to form not only in capillary-like structures but also as lumens	Measure cell movement, cell morphology, cell-cell adhesion, and cell polarity	Migration of endothelial cells can be observed and analyzed easily in both horizontal and vertical directions Require a longer time to run (5-15 days), difficult to view and quantify, and require histological techniques or confocal microscopy Care should be given to the width of the matrix that might result in additional difficulties in diffusion of nutrients and oxygen	[49, 50]
	3D spherical-shaped assay or microcarrier or microtissue assay	Microcarrier beads are used for the growth of endothelial cells into a confluency rate, which is implanted into fibrin gels	The migration of endothelial cells that form capillary-like structures can be observed and analyzed quantitatively	Avoid the endothelial cell detachment problem Microcarriers may descend to the base of the gel and limit the breadth of the gel	[51, 107]

Endothelial-mural cell coculture assays	Direct contacting assay	To determine the effects of endothelial cells on mesenchymal cell differentiation	Quantify the straight effects of one cell type	Simple and easy to analyze, but lacks the effect of paracrine factors that can be released by one cell type	[56, 108]

**Table 2:** *In vivo* techniques of angiogenesis.

*In vivo* techniques	Type of methods	Biological scope	Assay reliability (quantitative or qualitative)	Pros/cons	References
Quantitative determination of tissue blood flow rate	Tissue blood flow rate	The process of delivering the arterial blood into the capillary beds within a scrupulous group of tissues	Measure the rate of delivery of an agent carried to the tissue by blood flow	More sensitive and acquires an appropriate pharmacodynamic endpoint	[109]
	Fluorescent DNA-binding staining Hoechst 33342	To measure perfused vascular volume as a fraction of the total tissue volume	Functional vessels appear as fluorescent halos after intravenous injection of the radioactive dye	Unable to discriminate between perfused vessels with different flow rates and lacks appropriate sensitivity	[68]
*In vivo* Matrigel migration and angiogenesis assay	Marine Matrigel plug assay	Measuring angiogenesis and anti-angiogenesis	Quantitative method	Gives the best results when testing putative antiangiogenic compounds	[72]
Corneal pocket assay	Corneal assay	Implanted into a micropocket produced in the cornea thickness to induce angiogenesis by vascular outgrowths from peripherally located limbic vasculature	Characterization of angiogenesis inducers, evaluation of angiogenesis inhibitors, interaction between different factors, and study of cellular, biochemical, and molecular mechanisms of angiogenesis	Simple assay, shows excellent reproducibility compared to other *in vivo* assays Lack of blood vessels	[82]
Other *in vivo* models	Mouse syngeneic models, human xenografts, transgenic mouse models, and mutagenesis-induced mouse models	To monitor the blood of the tumor in real time and accompanying cellular events that coincided with pharmacodynamic endpoints	—	—	[88]

**Table 3:** Computational techniques of angiogenesis.

Computational techniques	Biological scope	Assay reliability (quantitative or qualitative)	Pros/cons	References
Phenomenological models	Neoangiogenesis can be used as an *in silico* tool to simulate the impact of a variety of drugs and regimen, thus allowing virtual screening of new compounds or comparison between different regimens	To predict how drugs will impact on neovasculature and tumor growth	Simple, provide better identification of parameters Lack of basic understanding of the underlying mechanisms	[92, 93]
PK/PD models	To study the direct impact of drugs on tumor vasculature	—	Remain theoretical	[96, 97]
